# Prevalence of dyslipidaemia and micronutrient deficiencies among newly arrived Afghan refugees in rural Australia: a cross-sectional study

**DOI:** 10.1186/1471-2458-14-896

**Published:** 2014-09-01

**Authors:** Mehdi Sanati Pour, Surabhi Kumble, Sarah Hanieh, Beverley-Ann Biggs

**Affiliations:** Tristar Medical Group, Mildura, 3500 Victoria Australia; Melbourne Academic Centre, at the Peter Doherty Institute for Infection and Immunity, University of Melbourne, Melbourne, 3010 Victoria Australia; Victorian Infectious Diseases Service, Royal Melbourne Hospital, Melbourne, 3050 Victoria Australia

**Keywords:** Dyslipidaemia, Micronutrient deficiencies, Afghan refugees, Rural Australia

## Abstract

**Background:**

Afghanistan is the 15^th^ least developed country in the world, with poor sanitation and high rates of infectious diseases and malnutrition. However, little is known about the health of young Afghan refugees resettling in Western countries.

**Methods:**

We used a cross-sectional study design to examine the health profile of newly arrived Afghan refugees presenting to a General Practice between 20^th^ April 2010 and 22^nd^ March 2013 in rural Australia. Data collected included information on nutritional status and prevalence of infectious diseases. Challenges associated with health screening in a General Practice setting in this population were also documented.

**Results:**

Data were available on 92 patients. Mean age of presentation was 22.6 years [SD 11.9], and the majority of patients were male (88%). Mean total cholesterol, LDL cholesterol, HDL cholesterol and triglyceride concentrations were 4.4 mmol/L (95% CI, 2.9–7.3), 2.6 mmol/L (95% CI, 1.3–4.4), 1.24 mmol/L (95% CI, 0.7–2.0) and 1.19 mmol/L (95% CI, 0.4–4.7) respectively, and dyslipidaemia (defined as elevated total or *low-density lipoprotein* (LDL) cholesterol levels, or low levels of high-density lipoprotein (HDL) cholesterol) was seen in 27.5% of patients. There was also a high prevalence of vitamin D and B12 deficiency (50% and 18%, respectively) in this cohort. Issues that impacted on provision and access to health care for refugees included cost, language barriers, patient mobility and mental health issues.

**Conclusions:**

Dyslipidaemia and micronutrient deficiencies are significant health issues in young recently settled Afghan refugees, and routine screening should be considered for this population.

## Background

Australia is the largest settlement country for refugees on a per capita basis [1]. In 2012, the Australian Government increased its humanitarian program to 20,000 places - a more than 40 per cent increase, and the largest increase in intake within 30 years [1]. Afghan refugees are expected to make up a large proportion of this intake in the foreseeable future. Worldwide, Afghans form the largest group of refugees [2], and the withdrawal of NATO troops from Afghanistan in 2014 is likely to cause further political instability in the country.

Afghans first migrated to Australia in the 1860s as employees, bringing camels to aid their exploration for resources. Another surge of migration has occurred in the last decade and between 2008–13, 12,063 Afghan migrants have settled in Australia [3]. The majority live in southern Australia, and are aged between 18 and 34 years [4].

Afghan refugees have unique health problems due to the political and socio-economic circumstances they have encountered in Afghanistan, and during their journey to Australia. The majority have fled war, violence and trauma and are dealing with grief, loss and forced separation from their homes [5]. Afghanistan’s health status is one of the worst in the world which reflects poor nutrition through the life cycle and communicable diseases, compounded by the lack of provision of adequate primary health care and public health programs across the country. The life expectancy at birth for both males and females is 48 years and the maternal mortality rate per 100,000 live births is 460 [6]. The prevalence of tuberculosis is 352 per 100,000 population [6]. A 2012 study of 1200 adults in Kabul showed that 31.2% are obese (body mass index(BMI) ≥ 30 kg/m^2^) with twice as many women affected as men, 38.1% are overweight, and obesity was a risk factor for hypertension and diabetes [7].

The Australian Society of Infectious Diseases recommends screening refugees within the first month after arrival for tuberculosis, malaria, blood-borne viral infections, schistosomiasis, helminth infection, sexually transmitted infections, and other infections (eg, *Helicobacter pylori)* as indicated by clinical assessment, and catch-up immunisations where appropriate [8]. However, testing for glucose, cholesterol and triglycerides and other risk factors for non-communicable diseases is currently ad hoc, with no clear guidelines for this refugee group.

An increasing number of refugees are settling in rural areas in Australia, either through a formal relocation program, for employment, or even to escape racism and violence [5]. At the end of June 2011, 28 370 Afghan-born individuals were living in Australia, and eighty percent of permanent migration was accounted for through Australia’s Refugee and Humanitarian Programme. Fifty four percent of refugees were male, and the median age was 29.5 years. The Programme has two components, offshore resettlement and onshore protection. Since 2011 there has been a significant increase in the number of asylum seekers arriving by boat [4]. While there have been some studies documenting the settlement experience in rural areas [5], little is known about the health needs of Afghan refugees, especially in those settling in these areas, including in Mildura, a rural city 580 km north west of Melbourne with a strong dependence on agriculture. This paper is a pilot study in this area that aims to identify the most prevalent health issues in newly arrived Afghan refugees settling in this rural area.

## Methods

This was a cross-sectional study. We performed a retrospective review of laboratory results that had previously been collected as part of routine clinical practice, on all Afghan refugees presenting to a busy general practice in Mildura. Newly arrived Afghan refugee patients who presented to a General Practice between 20/4/2010 and 22/3/13 were included in the study. All refugees had previously spent time in various detention centres in Australia, mainly on Christmas Island.

As part of the initial refugee health screening consultation, routine laboratory tests included fasting glucose, cholesterol, thyroid stimulating hormone (TSH), malaria film and immune-chromatographic test, vitamin D levels, haemoglobin, serum ferritin and vitamin B12 concentration, stool microscopy and culture for gastrointestinal pathogens, serology for schistosomiasis, strongyloides, hepatitis B, hepatitis C, syphilis, rubella, and urinary PCR for *N. gonorrhoea and C. Trachomatis.* Barratt and Smith Pathology performed the laboratory tests (http://www.bsp.com.au), and standard reference ranges were used. Tests for schistosomiasis and strongyloides were referred to the Victorian Infectious Disease Reference Laboratory, Melbourne.

The treating GP (MSP) identified patients meeting the inclusion criteria and entered demographic information from the medical records into an excel database. Laboratory results were provided by the laboratory in an excel spreadsheet. The data was cleaned and checked for missing entries and inconsistencies, and entered into a Stata, Version 13 (StataCorp, College Station, TX, USA) for analysis. Categorical data are presented as percentages with frequency, and continuous data are presented as mean and standard deviation (SD). The significance of the difference in proportions was tested by means of *P* values calculated on χ2 distributions and continuous data using the student t-test.

Conditions were defined as follows: elevated fasting blood glucose: glucose > 7 mmol/L; anaemia: haemoglobin <130 g/L for men, haemoglobin <120 g/L for women; iron deficiency: serum ferritin <15ug/L; vitamin B12 deficiency: serum vitamin B12 < 150 pmol/L; abnormal TSH: serum TSH <0.3U/ml or >5.0 U/ml; abnormal lipid levels: total cholesterol ≥5.5 mmol/L, HDL < 1 mmol/L, LDL > 3.5 mmol/L, Triglyceride > 2.0 [9]. Dyslipidaemia was defined as elevated total or low-density lipoprotein (LDL) cholesterol levels, or low levels of high-density lipoprotein (HDL) cholesterol. Vitamin D deficiency was categorised as either: mild (30 to 49 nmol/L), moderate (12.5 to 29 nmol/L) or severe (<12.5 nmol/L) [10]. Exposure to a infection (STI) was defined as positive urinary PCR for *N.gonorrhoea or C.Trachomatis.* Rubella immune was defined IgG > 10 IU/ml, and hepatitis B immune as hepatitis B surface antibody positive (HepBsAb), surface antigen negative (HepBsAg), and core antibody negative (HepBcAb); or HepBsAb positive, HepBsAg negative and HepBcAb positive; or HepBsAb negative, HepBsAg negative, and HepBcAb positive.

A semi-structured interview was also conducted with the treating GP in the practice by a member of the research team, to identify challenges associated with initial health screening in this population. These observations are presented in Table 1. Informed consent was obtained prior to undertaking the interview.

**Table 1:** **Observations around challenges of initial health screening in a refugee population in a rural general practice**

Questions	Observations from treating general practioner
Challenges to undertaking full health screen in Mildura, Victoria	• Cost of screening tests
	•Lack of information available from previous screening
	•Language barriers
	•Lack of understanding of the health system
	•Lack of trust of health professionals
	•Patient mobility
Prevalent mental health issues	•Anxiety
	•Depression
	•Post-traumatic stress disorder
Strengths of the refugee health program at the clinic	•GPs and reception staff of various ethnic backgrounds (e.g. Dari, Turkish, Persian, Tamil)
	•Bulk-billing of all patients
	•Extended opening hours (8am-8pm, 7 days/week)
	•Integrated mental health system in clinic
	•Access to refugee health and infectious diseases specialists
Other issues with refugee health related to a rural setting	•Anxiety due to family separation

As this was a retrospective review of laboratory results previously collected as part of routine clinical practice, written informed consent was not obtained from patients. Ethics approval was obtained from the Royal Melbourne Hospital Human Research Ethics Committee.

## Results

**Figure 1:**
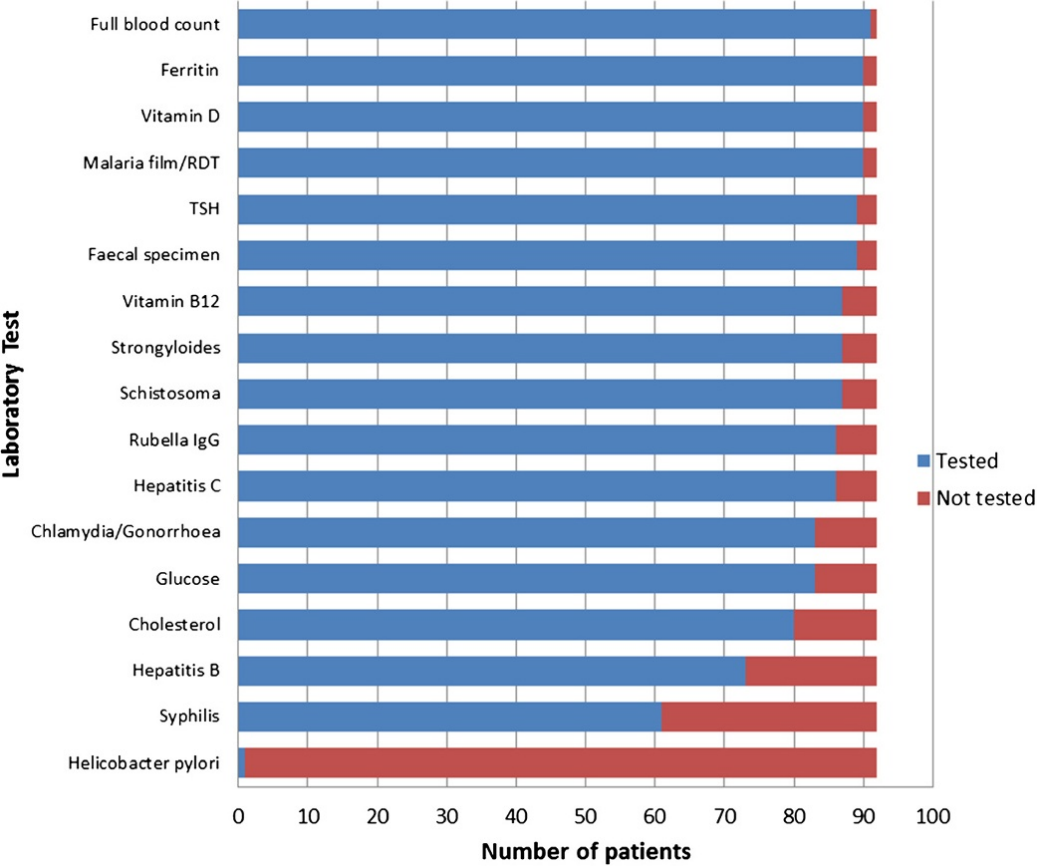
**Most frequent laboratory tests performed by GP practice on newly arrived Afghan refugees to Mildura, Victoria, 2010–2013.**

Ninety-two patients presented to the clinic for a refugee health assessment during the thirty-five month study period. Table 2 depicts the baseline characteristics of the patients. The mean age of presentation was 22.6 years [SD 11.9], and the majority of refugees were male (88%). The majority of patients arrived by boat in Australia, and were detained in Christmas Island, or had spent time in detention or in community detention in Indonesia before being accepted as refugees in Australia. Patients were generally held in detention centres between 12 months and 3 years before being released into the community.The most frequent laboratory tests performed are presented in Figure 1.

**Table 2:** **Baseline characteristics of 92 newly arrived Afghan refugees seen in a general practice clinic in Mildura, Victoria 2010-2013**

Characteristic	Values ^1^
**Demographic**	
**Age (years)**	22.6 [11.9]
0-10	19 (20.7)
11- 21	28 (30.4)
22-32	25 (27.2)
33-43	15 (16.3)
>43	5 (5.4)
**Gender**	
Female	11 (12.0)
Male	81 (88.0)
Weight (kg)^2^	63.3 [18.2]
Height (cm) ^2^	162.9 [20.2]
**Body mass index (kg/m** ^**2**^ **)**	22.9 [3.93]
Underweight (BMI<18 kg/m^2^)	9 (12)
Normal (BMI 18-25 kg/m^2^)	43 (57.3)
Overweight (BMI >25 kg/m^2^)	23 (30.7)

^1^ Values are mean [standard deviation] or number (%).

^2^ Data missing on 17 patients.

Overall, vitamin D deficiency (50%) and abnormal cholesterol levels (27.5%) were the most commonly identified health issues in these young Afghan refugees. Table 3 shows the prevalence of common health issues identified, stratified by gender.

**Table 3:** **Five most common problems identified in newly arrived Afghan refugees in general practice, Mildura Victoria, 2010–2013**

	Total number (%)	Male number (%)	Female number (%)	Chi squared
Vitamin D deficiency (vitamin D <50 nmol/L)	45 (50.0)	35 (44.3)	10 (90.9)	8.39*
Dyslipidaemia	22 (27.5)	20 (27.8)	2 (25)	0.03
B12 deficiency (serum vitamin B12 < 150 pmol/L	16 (18.4)	15 (19.7)	1 (9.1)	0.73
Giardia	10 (11.2)	8 (10.1)	2(20)	0.87
Anaemia (Hb < 130 g/L male Hb < 120 g/L female)	6 (6.6)	4 (5)	2 (18.8)	2.72

*P < 0.05.

### Dyslipidaemia

**Figure 2:**
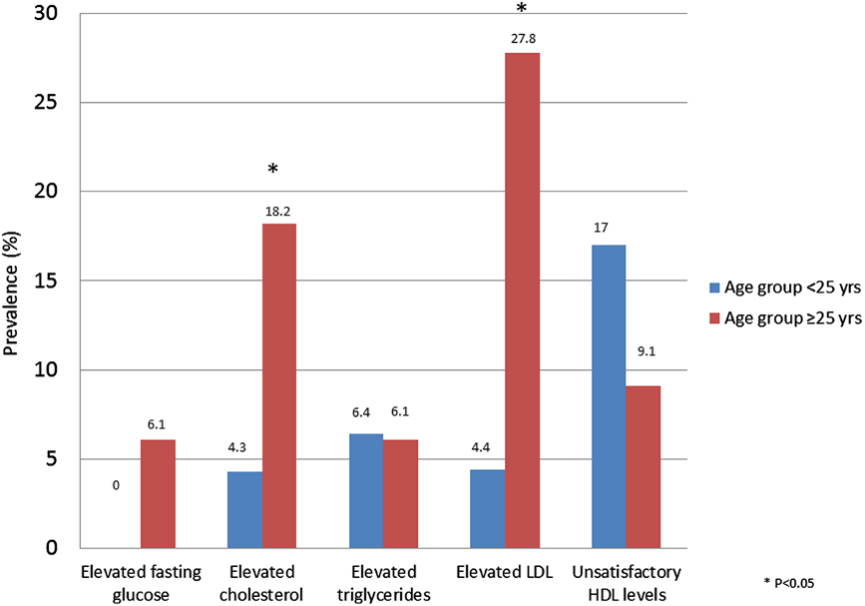
**Prevalence (%) of newly arrived Afghan refugee patients with dyslipidaemia or elevated glucose levels in Mildura, Victoria 2010–2013.**

Mean total cholesterol, LDL cholesterol, HDL cholesterol and triglyceride concentrations (TG) were 4.4 mmol/L (95% CI, 2.9–7.3), 2.6 mmol/L (95% CI, 1.3–4.4), 1.24 mmol/L (95% CI, 0.7–2.0) and 1.19 mmol/L (95% CI, 0.4–4.7), respectively. The prevalence of elevated total cholesterol, LDL and TG, and decreased HDL-C concentrations were 11.3%, 13.9%, 6.3% and 13.8%, respectively. Over one quarter of patients (27.5%) had dyslipidaemia (defined as elevated total or LDL cholesterol levels, or low levels of HDL cholesterol). Elevated total cholesterol (χ2 = 6.38 P = 0.01) and LDL-cholesterol (χ2 = 7.99 P = 0.01) levels were significantly associated with age (Figure 2).

### Other chronic disease risk factors

More than twenty percent of patients were smokers and 30% of patients were classified as overweight (BMI > 25 kg/m^2^). Prevalence of elevated fasting blood glucose in this population was low (2.5%) (Table 4).

**Table 4:** **Patients with chronic disease risk factors by age group**

Risk factor	Total	Age >25 years	Age <25 years	Chi squared
Smoker	18 (23.7)	28.0	21.6	0.39
Overweight (BMI > 25 kg/m^2^)	23 (30.7)	12 (24)	11(44)	3.14
Hypertension (systolic BP ≥ 140 mmHg or diastolic BP ≥ 90 mmHg)	16 (21.1)	8 (15.7)	8 (32)	2.67
Dyslipidaemia	22 (27.5)	12 (36.4)	19 (21.3)	2.21
Elevated fasting blood glucose	2 (2.53)	2 (6.9)	0(0)	2.86

### Nutritional deficiencies

**Figure 3:**
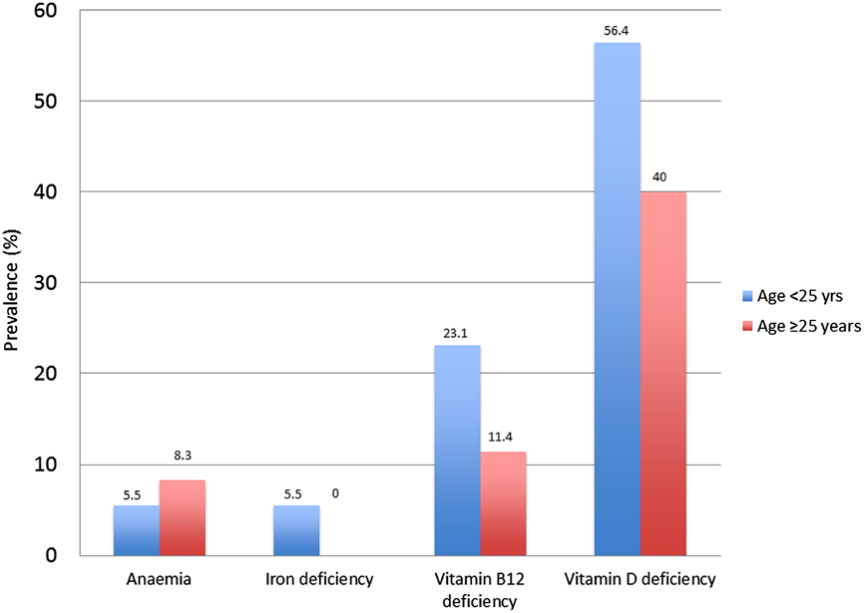
**Nutritional deficiencies (%) identified in newly arrived Afghan refugee patients in Mildura, Victoria, 2010–2013.**

Nutritional deficiencies stratified by age (under or over 25 years) are presented in Figure 3.

Vitamin D deficiency was the most common deficiency. Mean vitamin D concentration was 49.6 nmol/L [14.7]. Mild vitamin D deficiency (30-49 nmol/L) was seen in 27.8% of patients, 21.1% had moderate deficiency (12.5 to 29 nmol/L), and <2% had severe deficiency (<12.5 nmol/L). The one case of severe vitamin D deficiency (<12.5 nmol/L) was in a 34 year old female patient. Vitamin D deficiency was more prevalent in female, compared to male patients (χ2 = 8.39 P = 0.03) The prevalence of vitamin B12 deficiency was 18.4%, and anaemia occurred in less than 10% of patients (Table 3).

### Infectious diseases

Giardia, schistosomiasis and strongyloidiasis were the most commonly identified infectious diseases. Eosinophilia was present in 7.7% of patients. No patients with eosinophilia were found to have a pathogenic stool parasite, or positive schistosomoma or strongyloides serology. No cases of malaria were documented.

### Access to health care

A number of other issues impacting on provision and access to health care for these refugees were identified through an in-depth semi-structured interview with the treating GP. Table 1 summarises the observations identified during the interview, including cost, language barriers, patient mobility and mental health issues.

## Discussion

Our results indicate that dyslipidaemia and vitamin D deficiency are common health issues in newly arrived Afghan refugees to rural Australia, and that language, cost and mental health issues are important considerations in provision and access to health care within this community. To our knowledge, this is the first study to document health issues in newly arrived Afghan refugees settling in Australia.

In our study, almost 30% of patients had an elevated total cholesterol, LDL, or inadequate HDL levels at initial presentation for health screening, despite the majority of study participants being young adults with a mean age of 23 years. This high rate of dyslipidaemia most likely reflects under-nutrition in early life, as well the transition of the Afghan population to a higher fat diet and sedentary life style in the last few decades. Increasing evidence suggests that under-nutrition during pregnancy and in the first two years of a child’s life may lead to disproportionate foetal growth and persisting changes in cholesterol metabolism, insulin responses to glucose, and other metabolic parameters, resulting in the programming of chronic diseases such as hypertension, coronary heart disease, and high cholesterol later in life [11–13]. In Afghanistan 20% of infants are born with low birth weight (birth weight <2500 grams), rates of stunting in the first 5 years of life are close to 60%, and 33.7% of children are underweight by 5 years of age [14, 15]. This is compounded by transitions in dietary patterns, with increases in saturated fat, cholesterol and dietary energy density, and a reduction in exercise levels in adolescence and young adulthood, the so-called double burden of malnutrition [16, 17]. Further research is needed to clarify whether the high rate of dyslipidaemia seen in these young adults is already present before leaving Afghanistan, or is a result of the migration process including dietary and exercise patterns in transit countries, such as Indonesia, and in Australian detention centres [18, 19].

The focus of refugee health in previous studies has mainly centred on infectious diseases, and there are limited studies with data on the prevalence of dyslipidaemia and non-communicable diseases in newly arrived refugees. Yun et al. found that half of the adult refugees (median age 31 years) screened on arrival in the United States had at least one chronic non-communicable disease (51.1%), and 9.5% had three or more non-communicable diseases [20]. Dodson et al. have demonstrated a high prevalence of hypercholesterolaemia (66%) in Southeast Asian refugees resettling in the United States (median age 48 years) [21]. It has also been demonstrated that refugees who have experienced extensive food deprivation or insecurity are more likely to engage in unhealthy dietary practices following migration [22]. Dyslipidemia in rural Australia has previously been demonstrated by Janus et al. in a non-refugee population, where hypercholesterolaemia was found to be 39%, highlighting the need for improved risk factor management for hypercholesterolaemia and implementation of preventive strategies at the primary care level in rural populations [23]. There is an increasing global prevalence of chronic non-communicable diseases, and they now account for 61% of all deaths and 46% of the burden of disease among low and middle income countries [24]. Our findings highlight the need for careful screening within this population, particularly as refugees transition out of the early settlement period, and experience a shift to Western dietary patterns, with increased intake of fat and carbohydrate rich foods [25].

Vitamin D deficiency was the most common nutritional deficiency seen in this population, as has been demonstrated in other refugee studies [26–28]. More than 90% of females were deficient, and it is likely that cultural practices are a major risk factor, including wearing concealing clothing and spending more time in doors with limited daily sun exposure. Vitamin D is essential for skeletal health and bone mineralization. Vitamin D deficiency is often asymptomatic, but may present with vague symptoms such as muscle pain [29]. Severe deficiency may result in rickets in children and osteomalacia in adults, and deficiency has also been associated with infectious and autoimmune diseases, cardiovascular diseases, diabetes mellitus, cancer, and adverse pregnancy outcomes [30–35]. Our findings highlight the importance of regular testing for vitamin D deficiency in refugee populations, and consideration of routine Vitamin D supplementation in at risk groups [36, 37].

We also found a relatively high prevalence of Vitamin B12 deficiency in this population, even though few were anaemic. Benson et al. (2010) have previously described low vitamin B12 levels (prevalence of 16.5%) among newly arrived refugees from Bhutan, Iran and Afghanistan [38]. Food insecurity in countries of origin, inadequate nutrition during the asylum-seeking process, lack of consumption of animal products, and barriers to accessing B12 rich foods, e.g. due to cost, may contribute to the high prevalence of vitamin B12 deficiency in this population [26]. Vitamin B12 deficiency may be difficult to detect clinically as it commonly manifests with vague neurological and generalised symptoms such as paraesthesia, irritability, personality change, memory impairment, depression, fatigue, loss of appetite and weight loss. Severe deficiency can also lead to serious adverse consequences including subacute combined degeneration of the spinal cord and increased risk of myocardial infection and stroke. Infants born to vitamin B12 deficient mothers may also be at risk of severe, irreversible neurological damage [26, 38–40]. Routine screening of B12 levels in this population is therefore important.

We documented a number of challenges observed in the provision of care to newly arrived Afghan refugees in this rural area including language, cost and understanding of the health system. Refugees who have previously resettled in similar areas may be useful in bridging these challenging language and cultural gaps (i.e. a ‘local champion’) [41]. Mental illness was also observed to be a significant health issue. Challenges to the provision of mental health care were noted to be a lack of refugee-specific mental health services in Mildura, as well as language and cultural differences. Effective prevention and management of mental health problems in newly arrived refugees in a primary care setting requires awareness of these issues [42].

Strengths of our study include the sample size, and the comprehensive screening undertaken. A limitation is the low number of females included (12%) due to the small number of Afghan women being settled in Mildura, however the male refugees in this study are generally representative of the overall Afghan community, as fifty four percent of Afghan born refugees accounted for through Australia’s Refugee and Humanitarian Programme are male.[4] A further limitation is the lack of access to other health information on participants such as liver ultrasound to assess fatty infiltration of the liver. However despite these limitations, our findings provide an important overview of the health status of newly-arrived Afghan refugees and potential barriers to care in a rural general practice setting. They will help to inform primary health care providers and policy makers, so that screening and provision of health care to Afghan and other refugees may be appropriately and effectively targeted.

## Conclusions

Dyslipidemia and micronutrient deficiencies are important health issues in newly-arrived Afghan refugees to rural Australia, and routine screening should be considered in this population. This is a pilot study in this area and further research should explore the prevalence and morbidity from non-communicable diseases in Afghan refugees, as well as factors to improve provision and access to health care in this growing population.

## Abbreviations

BMI: Body mass index
LDL: Low density lipoprotein
HDL: High density lipoprotein
TG: Triglyceride.
